# Attachment Characteristics Among Women Victimized in No, One, and Multiple IPV Relationships: A Case–Control Study

**DOI:** 10.1177/1077801220981157

**Published:** 2021-02-11

**Authors:** Elisabeth Christie Ørke, Stål Bjørkly, Mariana Dufort, Solveig Karin Bø Vatnar

**Affiliations:** 1 Oslo University Hospital, Norway; 2 Molde University College, Norway; 3 Karolinska Institute, and Centre for Psychiatry Research, Stockholm, Sweden

**Keywords:** intimate partner violence (IPV), multiple partners (MP), attachment, childhood emotional abuse

## Abstract

This cross-sectional study compared attachment characteristics among women victimized by intimate partner violence (IPV) in no, one, and multiple relationships (*N* = 154). Results indicated that compared with the nonvictimized, victimized women had increased likelihood of higher attachment avoidance. Compared with women victimized in one relationship, women victimized in multiple relationships had higher likelihood of higher attachment anxiety scores. Adjusting for childhood adversities, childhood sexual abuse was an independent risk factor for IPV. Childhood emotional abuse mediated the association between attachment anxiety and IPV victimization in multiple relationships in particular. Attachment theory appeared useful for better understanding women’s vulnerability for multiple violent relationships.

## Introduction

Intimate partner violence (IPV) is a serious, heterogenic, and complex issue associated with significant health, social, and economic costs to individuals, families, and society (Cattaneo & Goodman, 2005; Cornelius & Resseguie, 2007; Costa et al., 2015; Mears, 2003; Park, 2016). IPV comprises physical and sexual violence, stalking, and psychological aggression (including coercive tactics) by a current or former intimate partner (Breiding, 2015). In contrast to other types of violence, IPV is commonly repetitive and tends to escalate in both frequency and severity along with the duration of the relationship (Cochran et al., 2011). Many women who have been subjected to IPV have experienced IPV in multiple relationships. In studies of IPV victimized female samples, 23–61% of the women had prior histories of IPV relationships (Dufort, Gumpert, & Stenbacka, 2013; Vatnar & Bjorkly, 2008). However, there is limited knowledge about factors that contribute to women having repeated experiences of IPV by successive partners (Smith & Stover, 2016). Accordingly, it is urgent to investigate victim-related risk factors for IPV by multiple partners (MP).

Although a perpetrator must be held accountable for the violence, focusing on only the perpetrator may distract attention from a possible vulnerability in some women for being victimized by MP. It is important to keep in mind that risk factors do not establish a causal relationship (Park, 2016) and that a victim is not responsible for the victimization; however, identification of empirically validated victim-related risk factors may help practitioners guide victims in decision making and safety planning (Cattaneo & Goodman, 2005) and inform the prevention of future IPV relationships.

Research on IPV victimization has predominantly been conducted on female victims in heterosexual relationships, focusing on adversities and vulnerability factors that may influence the risk of revictimization. Although rates of IPV were found to be similar between women and men (Straus, 2011), there were substantial differences in the consequences reported. Female victims were more likely to suffer more severe consequences than males were (Archer, 2000; Askeland, 2015; Caldwell et al., 2012; Nybergh et al., 2013; Stöckl et al., 2013; Wathen & MacMillan, 2003). Johnson and colleagues proposed specific types of IPV that were differently gendered regarding mutuality. Situational couple violence (SCV), referring to isolated violent acts commonly caused by specific conflicts, was more likely to be mutual. Intimate terrorism (IT), referring to violent coercive control over one’s partner, was more likely to be perpetrated by men toward women (M. P. Johnson, 2011; Johnson & Leone, 2005). Exploring this typology and gender, Jasinski and colleagues (2014) reported that women were not more likely than men to be the victims of IT, but female sufferers of IT were significantly more likely than males to be injured from the violence, to attempt to leave their husbands, and to report desistance (Jasinski et al., 2014). Others reported that victimized women experienced more physical and emotional impairment than men did, and sought help more frequently than male IPV victims did. Victimized women also reported more fear and intimidation than men did when their partner initiated violence (Askeland, 2015; Wathen & MacMillan, 2003). Globally, the proportion of murdered women killed by a partner was six times higher than the proportion of murdered men killed by a partner (Stöckl et al., 2013). Based on this, the present study focused on female victims of IPV.

Bell and Naugle (2008) suggested a contextual framework for conceptualizing IPV episodes. It included distal, static, and proximal antecedents; motivating factors; behavioral repertoire; discriminative stimuli; verbal rules; and IPV consequences.

Expanding on an integrative model, Winstok (2007) proposed an interactional approach to the study of IPV. From initially addressing the parent–child relationship, attachment theory has been suggested as useful in addressing couple relationships and conflict (Cassidy & Shaver, 2016). Adult attachment style is significantly associated with fundamental components of romantic relationships, including the capacity for intimacy, partner caretaking and support, sexual behavior, conflict management, and relational aggression (Riggs, 2010). Hence, there is both a developmental and a social attachment approach in attachment theory. Within the contextual, interactional framework both were relevant to this study of victim-related risk factors for IPV by MP. The present study is informed by differential expressions of adult attachment among groups of IPV victimized adult women. Childhood adversities are central in attachment theory and controlled for in the statistical analyses.

### Victim-Related Risk Factors for IPV

Studies on generic IPV reported anxious (Bond & Bond, 2004; Lewis et al., 2017; McClure & Parmenter, 2017; Ponti & Tani, 2019; Shechory, 2013), avoidant (Ponti & Tani, 2019; Shechory, 2013; Weiss et al., 2011; Wekerle & Wolfe, 1998), and preoccupied attachment characteristics (Henderson et al., 2005) as risk factors for IPV victimization. According to a systematic review (Velotti et al., 2018), most studies on attachment and IPV victimization focused on physical or psychological IPV, and the association between specific attachment dimensions and such specific types of violence victimization was inconclusive.

Other than attachment issues, studies reported childhood risk factors for victimization of IPV. These were in particular psychological abuse/maltreatment (Cascio et al., 2017; McClure & Parmenter, 2017; Reyome, 2010; Wekerle & Wolfe, 1998), sexual (Ørke, Bjørkly, & Vatnar, 2020) and physical abuse (e.g., Barrios et al., 2015; Cascio et al., 2017; Hetzel-Riggin & Meads, 2011), exposure to parental abuse (Ehrensaft et al., 2003; Hetzel-Riggin & Meads, 2011; Krishnan et al., 2001), and peer victimization (Ørke et al., 2020).

Victimization and revictimization may not be the same. Avoidant attachment was reported as a risk factor for revictimization (Kuijpers et al., 2012b). Smith and Stover (2016) reported that attachment anxiety moderated the relationship between traumatic experiences and IPV revictimization. Reported childhood risk factors for revictimization of IPV were sexual and physical abuse (Coid et al., 2001; Stroem et al., 2019) and exposure to parental abuse (Trickett et al., 2011). Reported adult risk factors for IPV revictimization were female angry and aggressive behavior and initiation of violence (Kuijpers et al., 2012a, 2012b, 2012c) and some resistance and coping strategies (Goodman et al., 2005; Iverson et al., 2013).

### Victim-Related Risk Factors for IPV by Multiple Partners

Most studies on IPV revictimization have not distinguished between revictimization by the same partner and revictimization by subsequent partners, which implies mixed and inaccurate results (Ørke et al., 2018). Risk factors related to recurrent violence within the cycle of a single violent relationship may not be the same as the risk factors related to IPV by MP. A victim at risk of repeated victimization within a relationship would need other interventions than a victim at risk of revictimization by MP. Therefore, it is important to study the specifics of different forms of revictimization.

A systematic literature review (Ørke et al., 2018) regarding revictimization of IPV by MP in particular indicated that IPV by MP was significantly associated with childhood domestic trauma, drug abuse, and IPV characteristics. Attachment style as a risk factor for IPV by MP was investigated in one study which reported that women who were unresolved in their attachment had increased risk of IPV by MP (Alexander, 2009). A classification of unresolved attachment regarding trauma or loss was based on the presence of uncorrected or unexplained lapses of discourse or reasoning (Alexander, 2009). Regarding posttraumatic stress disorder and personality disorders, the results were mixed and inconclusive, and depression did not appear as a salient risk factor for IPV by MP. A recent study compared parental psychological and physical violence and childhood emotional neglect and abuse and concluded that among these, childhood emotional abuse was a risk factor for IPV by MP (Ørke et al., 2020).

According to the reviewed literature, both attachment issues and childhood adversities were suggested as risk factors for IPV by MP. Other studies reported an association between attachment issues and childhood adversities: childhood maltreatment (physical, sexual, or emotional abuse) and neglect (disengaged and extremely insensitive parenting) were consistently found to increase the rate of children’s attachment insecurity (Mikulincer & Shaver, 2016) and were associated with IPV by MP (Ørke et al., 2020). In the present study, we investigated attachment anxiety and avoidance while adjusting for significant childhood adversities. This was guided by the contextual, interactional framework and attachment theory.

### Attachment Theory and Victim-Related Risk Factors for IPV and IPV by MP

Early positive experiences of parental caregiving play a causal role in the formation of a child’s stable sense of attachment security. Atypical parental behavior can influence the development of different types of attachment insecurity. These effects tend to persist over time and contribute to attachment patterns during adolescence and adulthood (Mikulincer & Shaver, 2016).

Research has shown that adult attachment style can be measured along two orthogonal dimensions: attachment anxiety and attachment-related avoidance (Brenner et al., 2021; Mikulincer & Shaver, 2016). According to Brennan et al. (1998), these two dimensions underlie virtually all self-report adult romantic attachment measures and appear crucial for capturing important individual differences in adult romantic attachment. Adults with secure attachment have low scores on both attachment anxiety and attachment avoidance and subsequently are more likely to be involved in healthy and stable romantic relationships (Hazan & Shaver, 1994). Secure attachment may serve as a buffer against the negative implications of adverse life events (Bowlby, 1969). Attachment anxiety involves excessive need for approval from others, fear of interpersonal rejection or abandonment, and distress when one’s partner is unavailable or unresponsive. Attachment avoidance, on the other hand, involves need for self-reliance and fear of interpersonal closeness (Cassidy & Shaver, 2016; Pedersen et al., 2015).

#### Attachment measurements

Adult attachment has been studied in two research traditions that apply somewhat different methodologies: the developmental approach and the social approach. Within the developmental approach, attachment styles are measured through adults’ narratives of their childhood experiences with caregivers (Bartholomew & Shaver, 1998). Within the social attachment approach, measurements of attachment styles are based on self-reports regarding qualities in current close relationships in adulthood (Pedersen et al., 2015). Some of the concepts have similar wording across measurements but are not identical (e.g., secure, preoccupied, dismissive-avoidant, and fearful-avoidant within the developmental approach, and secure, anxious, and avoidant within the social approach). Different aspects of the concepts are weighted differently in the various measurements. Adult attachment style has been conceptualized and measured both in terms of types and dimensions. Mikulincer and Shaver (2016) concluded that adult attachment styles assessed with self-report measures were best characterized by dimensional measures.

As described above, studies have found an association between both attachment anxiety and attachment avoidance and IPV in general, but research regarding this association and IPV by MP is scarce, and further research is needed. In the present study, we investigated attachment differences between IPV victimized and nonvictimized women and between women victimized by one and by multiple partners.

The research questions were the following:

**Research Question 1:** Are women victimized by IPV from one or multiple partners different from women with no IPV relationships regarding adult attachment characteristics, adjusting for childhood adversities and sociodemographic variables?
**Research Question 2:** Are women victimized by IPV from one partner different from women victimized by IPV from multiple partners regarding adult attachment characteristics, adjusting for childhood adversities and sociodemographic variables?

## Method

### Design

This study was a part of a cross-sectional study with two groups of help-seeking, IPV victimized women and a control group of help-seeking women not IPV victimized.

### Participants

Participants were recruited according to the following criteria:

- Participants were at least 18 years old.
- They had contact with police, family counseling, women’s shelter, or the Alternative to Violence treatment center (ATV) for IPV or other family problems.
- They were in, or had recently been in, an intimate relationship that had lasted at least 6 months (index relationship).
- They held either Norwegian citizenship or a residence permit.
- They had sufficient language fluency to understand the information letter and to make an appointment on the phone.
- They had experienced IPV either within the last 3 years or not at all.

All participants in the control group were recruited from family counseling offices. These participants shared with the study groups the characteristics of being adult women experienced with a recent intimate relationship and seeking help for intimate partner-related problems.

Twenty-three local offices in rural and urban areas across Norway recruited participants to the study. There were 307 women who were invited to participate. Among these, 32 did not meet inclusion criteria when this was controlled on the phone, and 91 declined to participate. Among the 184 who consented to participate, 8 became unreachable, 16 withdrew from the study, and 6 fell ill; 154 were included in the final sample. The total sample (*N* = 154) consisted of 36.4% (*n* =56) recruited from family counseling offices, 35.1% (*n* = 54) from shelters, 24% (*n* = 37) from ATV, and 4.5% (*n* = 7) of participants were recruited from the police. In five interviews (3.2%), a professional interpreter was hired.

The participants were between the ages of 20 and 69 (*M* = 39.85, *SD* = 10.28) and had a history of 1–13 intimate relationships (*M =* 2.97, *SD* = 1.824). There were women with no IPV relationships (31.2 %, *n* = 48), women with one IPV relationship (35.7%, *n* = 55), and women with multiple IPV relationships (33.1%, *n* = 51). Among the latter, the range was from two (62.7%, *n* = 32), three (23.5%, *n* = 12), four (7.8%, *n* = 4), five (3.9%, *n* = 2) to six IPV relationships (2%, *n* = 1). Ten participants were in an IPV relationship at the time of the interview. The index relationship had a mean duration of 10.5 years (*SD* = 8.9). Most women (85.7%, *n* = 132) were native Norwegians. There were 14.3% (*n* = 22) immigrants in the sample, and 24.7% (*n* = 38) of all the women had an immigrant partner in their index relationship. Nine of 10 women were mothers, and they had between one and six children (*M =* 2.29, *SD* = 1.030). Years of completed education ranged from 7–24 years, and the mean was 1–2 years above high school completion (*SD* = 3.282). More than half of the sample (55.8%) were employed, 13% had work assessment allowance, and 10.4% received disability benefits. Significant sociodemographic and contextual group differences among women in no, one, and multiple relationships are listed in Table 1.

**Table 1.** Sociodemographic and Contextual Group Differences Among Women With No (0IPVR), One (1IPVR), and Multiple IPV Relationships (2IPVR).

Variable	0IPVR (*n* = 48)	1IPVR (*n* = 55)	2IPVR (*n* = 51)	Total (*N* = 154)	1IPVR + 2IPVR vs. 0IPVR	1IPVR vs. 2IPVR
	% (*n*)	% (*n*)	% (*n*)	% (*n*)	*p*	*p*
Immigrant	8.3 (4)	25.5 (14)	7.8 (4)	14.3 (22)	.155	.016
Immigrant partner	4.2 (2)	40.0 (22)	27.5 (14)	24.7 (38)	<.001	.173
Work/income					.022	.014
Employed	77.1 (37)	50.9 (28)	41.2 (21)	55.8 (86)		
Student	6.3 (3)	12.7 (7)	3.9 (2)	7.8 (12)		
Unemployed	4.2 (2)	10.9 (6)	3.9 (2)	6.5 (10)		
Disability benefits	4.2 (2)	3.6 (2)	23.5 (12)	10.4 (16)		
Retired	2.1 (1)	0.0 (0)	2.0 (1)	1.3 (2)		
Other	2.1 (1)	9.1 (5)	3.9 (2)	5.2 (8)		
Work assessment allowance	4.2 (2)	12.7 (7)	21.6 (11)	13.0 (20)		
Mother	97.9 (47)	87.3 (48)	86.3 (44)	90.3 (139)	.031	.879
No confidants	0.0 (0)	9.3 (5)	9.8 (5)	6.5 (10)	.028	.900
Considers partner	87.5 (42)	81.8 (45)	52.9 (27)	74.0 (114)	.010	.001
Language challenges	2.1 (1)	18.2 (10)	8.0 (4)	9.8 (15)	.030	.125
Interpreter	0.0 (0)	9.1 (5)	0.0 (0)	3.2 (5)	.126	.027
Education (years) (*M*/*SD*)	16/2.60	15.49/3.36	13.41/3.26	14.96/3.28	<.001	.002
Length of relationship (years) (*M*/*SD*)	14.48/9.24	10.83/8.86	6.42/6.50	10.51/8.85	<.001	.003

*Note.* The Mann–Whitney *U* test was used to test for possible group differences for variables with nonparametric score distributions for two independent groups. The Pearson chi-square test was used for nominal data and unrelated groups. Age, age of partner, age at the initiation of first intimate relationship, time lapse since last relationship, and whether the victimized was presently in a violent relationship were tested with nonsignificant results. IPVR = intimate partner violence relationship.

### Measures

#### Attachment characteristics

Among commonly used self-report measures investigating attachment in the adult relationship, the questionnaire Experiences in Close Relationships (ECR) developed by Brennan et al. was reported to have the best psychometric properties (Brennan et al., 1998; Fraley et al., 2000). Many studies confirmed high construct and criterion validity (e.g., Cassidy & Shaver, 2016; Mikulincer & Shaver, 2016). The Norwegian version was reported to be psychometrically adequate in a general population of 30- to 45-year-old adults (Olssøn et al., 2010).

The ECR Norwegian validated version (ECR-N) (Olssøn et al., 2010) is a 36-item questionnaire comprising the following two subscales of 18 statements each: Attachment avoidance (labeled *Avoidance* in the tables: e.g., “I prefer not to show how I feel deep down”) and Attachment anxiety (labeled *Anxiety* in the tables: e.g., “I worry about being abandoned”). One study reported that exploratory factor analysis of the ECR indicated five subfactors of 4–6 items each, comprising two different aspects of Attachment avoidance and three aspects of Attachment anxiety: Avoidance of closeness, Uncomfortable with openness, Separation frustration, Anxiety for abandonment, and Frantic desire for closeness (Five-Factor Model, ECR-FF) (Pedersen et al., 2015). Respondents were asked to indicate how they, in general, experience romantic relationships, referring not only to their most recent but also to their prior romantic relationships.

Each statement was scored on a 7-point Likert-type scale, from 1, *not true at all*, to 4, *neutral*, to 7, *totally true*. The measures were derived by computing the mean of the 18 items for Attachment avoidance and Attachment anxiety, and the mean of the 4–6 items for each of the suggested five subfactors. Within the range from 1–7, higher scores indicated higher levels of Attachment anxiety and Attachment avoidance (Olssøn et al., 2010). Mean score in the Norwegian normative female population was 2.55 for Avoidance and 2.75 for Anxiety (Olssøn et al., 2010). Categories of high and low Attachment anxiety and high and low Attachment avoidance were computed using the mean of the Norwegian normative female sample (Olssøn et al., 2010) as the cutoff score, “high” being equal to or greater than the cutoff. The categories were analyzed initially for descriptive purposes but not in advanced analyses, because power and precision are lost when categories rather than continuous scales are used (Brennan et al., 1998; Mikulincer & Shaver, 2016). Both subscales were reported to exhibit high internal consistency reliability (Alonso-Arbiol et al., 2008).

#### Childhood adversities

Our modified version of the UngVold2015 (Mossige & Stefansen, 2016) covered childhood adversities like various acts of violence between, and from, the parents, frequency of Peer victimization (six scaled statements) and prevalence of Childhood sexual abuse (unwanted touching, attempted or forced penetration by someone before age 16; nine statements). Three parts of the Childhood Trauma Questionnaire (CTQ-SF) (Bernstein et al., 2003; Dovran et al., 2013) were applied through UngVold2015: frequency of physical neglect, emotional neglect, and emotional abuse. Childhood emotional abuse included endorsement of five scaled statements like “I thought my parents wished I never were born”; “I felt someone in the family hated me”; “As I see it, I was subjected to psychological maltreatment.”

#### Sociodemographic and contextual variables

The intimate relationship of interest for research (index relationship) was the most recent violent relationship for victimized participants and the most recent relationship for nonvictimized participants. The following variables about this relationship, this partner, and this participant were recorded. Age, Age of partner, whether the participant considered herself and her partner as of ethnic Norwegian origin or immigrant with/without Norwegian citizenship (Immigrant; Immigrant partner), whether the participant was a mother (Mother), years of completed education (Education), work/income situation (Employed including sick-leave, student, unemployed, disability benefit recipient, retired, work assessment allowance, or other), whether she had anybody to confide in (No confidants), age at the initiation of first intimate relationship, time lapse since last relationship (in months), length of recent relationship (in months; Length of relationship, transformed to years only for Table 1 to ease the reading), and whether the participant presently was in a violent relationship.

One question was developed by the current research group to test a clinical hypothesis regarding how much time the participants generally spend on considering a new partner: “I take my time when I choose a new partner.” The response (Considers partner) was computed as No (*not true*) and Yes (*true* or *partly true*) and analyzed separately among the sociodemographic control variables.

To explore reliability aspects in the participant’s answers, the following contextual variables were registered: Whether the participant and the researcher experienced some/considerable language challenges during the interview (Language challenges), and whether a professional interpreter conveyed the interview questions and answers (Interpreter).

### Procedures

The researchers cooperated with leaders of the nationwide agencies of women’s shelters, ATV, the police, and the family counseling agency in Norway to recruit participants to the study. The following procedures were followed: (a) The initial recruitment of participants was conducted by agency personnel by presenting an information letter to their female users, either in person or by phone; (b) after receiving written consent and contact information, the researcher sought contact with those recruited to discuss aspects of their participation in the study; and (c) the participating women came to a face-to-face interview with the same researcher, a female clinical psychologist, at the local recruitment or researcher’s office. Women who were not fluent in the Norwegian language were informed that a professional interpreter could be hired for the interview.

Women were included regardless of the sex of their partner. There was no economic incentive for participation, but a refund for public transport was offered. All participants were given a sheet with the answer alternatives for the questions. Time breaks were used when needed. The researcher registered the answers by hand in the codebook. The interviews lasted approximately 2 hours. The 154 interviews were carried out between March 2018 and January 2019.

#### Dependent variables

Violent relationship or violent partner was the unit of analysis. The women were recruited to the designated research category according to the definition of physical, psychological, and sexual violence in the information letter (Breiding, 2015). They were asked (both on the phone and, initially, in the interview) in how many intimate relationships they had experienced violence victimization. According to self-report, the participants were grouped into one of the following three research categories of intimate partner violence relationships (IPVR):

- 1IPVR, women who had experienced violence from one intimate partner within the last 3 years.
- 2IPVR, women who had experienced violence from an intimate partner within the last 3 years and in at least one previous intimate relationship.
- 0IPVR, women who currently or lately had an intimate relationship but never had been victims of IPV (control group).

### Statistical Analyses

Univariate and bivariate analyses were conducted to compare the subgroups—(a) women with no IPV relationships (nonvictimized) compared with women with one and multiple IPV relationships (victimized), and (b) women with one IPV relationship (1IPVR) compared with women with multiple IPV relationships (2IPVR)—and to inform the selection of variables to be included in the multivariate analysis. Multivariate logistic regression analyses were used to examine group differences associated with victimization and with victimization in one or multiple IPV relationships.

The stepwise options recommended for logistic regression for small samples were used (Altman, 1991; Pallant, 2010). Step 1: Initial comparisons of the groups were carried out by simple descriptive cross-tabulations with Pearson chi-square for categorical and nominal variables. For continuous variables, we used *t*-tests for independent samples (Step 1, Tables 1 and 2). Nonparametric tests were used in case of skewed distribution. In the first multivariate logistic regression analyses (Step 2), sociodemographic and contextual variables with significant *(p* ≤ 0.05) or trend (*p* ≤ 0.10) in bivariate analyses were adjusted for other significant or trend differences within the same category. The significant or trend attachment variables from Step 1 were forwarded to Step 3 where each of them was tested in a separate multivariate logistic regression model adjusted for all remaining sociodemographic and contextual group differences from Step 2 (Model a). In Step 4, we adjusted for childhood adversities, which were found as risk factors in a previous part of this study (Ørke et al., 2020). In two extended models, we adjusted for interaction effects between the attachment factor and each of the childhood adversities. Only models with significant attachment variables are presented in the tables.

**Table 2.** Mean Scores of Attachment Characteristics Among Women With No (0IPVR), One (1IPVR), and Multiple IPV Relationships (2IPVR), Measured by Experiences in Close Relationships.

Variable	0IPVR (*n* = 48)	1IPVR (*n* = 54)	2IPVR (*n* = 50)	TOTAL (*N* = 152)	0IPVR vs. 1IPVR + 2IPVR	1IPVR vs. 2IPVR
	*M, SD*	*M, SD*	*M, SD*	*M, SD*	*p*	*p*
Anxiety	3.32, 1.09	3.49, 1.10	3.92, 0.97	3.58, 1.08	.048	.041
Avoidance	2.46, 0.89	3.37, 1.14	3.77, 1.21	3.21, 1.21	<.001	.086
Avoidance of closeness	2.37, 1.03	3.20, 1.24	3.70, 1.40	3.10, 1.34	<.001	.059
Uncomfortable with openness	2.33, 1.04	3.18, 1.31	3.59, 1.47	3.04, 1.38	<.001	.137
Separation frustration	3.72, 1.25	3.76, 1.18	3.89, 1.11	3.79, 1.18	.631	.553
Anxiety for abandonment	3.26, 1.68	3.44, 1.61	4.16, 1.60	3.62, 1.67	.071	.025
Frantic desire for closeness	2.85, 1.30	3.18, 1.25	3.56, 1.16	3.20, 1.26	.024	.118

*Note.* Independent samples *t*-test. Range = 1–7. IPVR = intimate partner violence relationship.

Suitability for multivariate logistic regression analysis was investigated by the Hosmer–Lemeshow test. Cox & Snell *R*^2^ and Nagelkerke *R*^2^ were used to estimate the proportion of explained variance in the multivariate models (Tables 3 and 4, Note). Statistical analyses were performed using the statistical program package SPSS, version 25. A conventional value of < 0.05 was used.

**Table 3.** Victimized (*n* = 105) Compared With Nonvictimized Women (Baseline) (*n* = 48) (Multivariate Logistic Regression Analyses).

Independent variables	Adjusted odds ratio	95% CI	*p*
Model 1 (*n* = 152)			
Avoidance	3.352	[2.036, 5.517]	<.001
Immigrant partner	18.568	[3.578, 96.373]	<.001
Length of relationship	0.993	[0.989, 0.997]	<.001
Model 2 (*n* = 152)			
Avoidance of closeness	2.214	[1.525, 3.213]	<.001
Immigrant partner	13.502	[2.794, 65.257]	.001
Length of relationship	0.994	[0.990, 0.998]	.003
Model 3a (*n* = 151)			
Uncomfortable with openness	2.700	[1.741, 4.188]	<.001
Immigrant partner	20.502	[3.977, 105.684]	<.001
Length of relationship	0.992	[0.988, 0.996]	<.001
Model 3b (*n* = 151)			
Uncomfortable with openness	2.656	[1.697, 4.157]	<.001
Childhood sexual abuse, prev.	2.784	[1.071, 7.236]	.036
Immigrant partner	22.494	[4.215, 120.025]	<.001
Length of relationship	0.993	[0.989, 0.997]	.001
Peer victimization, freq.			ns

*Note.* Multivariate logistic regression, forward stepwise (Wald). Model 1: Cox & Snell *R*^2^ = .347, Nagelkerke *R*^2^ = .486, Hosmer and Lemeshow test = .856. Model 2: Cox & Snell *R*^2^ = .294, Nagelkerke *R*^2^ = .412, Hosmer and Lemeshow test = .966. Model 3a Cox & Snell *R*^2^ = .324, Nagelkerke *R*^2^ = .454, Hosmer and Lemeshow test = .212. Model 3b: Cox & Snell *R*^2^ = .344, Nagelkerke *R*^2^ = .483, Hosmer and Lemeshow test = .149. Prev. = prevalence; freq. = frequency; CI = confidence interval.

**Table 4.** Women With Multiple IPV Relationships (*n* = 50) Compared With Women With One IPV Relationship (Baseline) (*n* = 54) (Multivariate Logistic Regression Analyses).

Independent variable	Adjusted odds ratio	95% CI	*p*
Model 4a (*n* = 104)			
Anxiety	1.776	[1.085, 2.909]	.022
Work/income			
Employed (baseline)			ns
Student			ns
Unemployed			ns
Disability benefits	17.578	[1.943, 159.055]	.011
Retired			^a^
Other			ns
Work assessment allowance			ns
Length of relationship	0.987	[0.980, 0.994]	<.001
Immigrant	0.136	[0.027, 0.694]	.016
Education			ns
Considers partner			ns
Model 4b (*n* = 104)			
Anxiety			ns
Childhood emotional abuse			ns
Anxiety × Childhood Emotional Abuse^b^	1.031	[1.010, 1.053]	.004
Work/income			
Employed (baseline)			ns
Student			ns
Unemployed			ns
Disability benefits	13.551	[1.603, 114.558]	.017
Retired			^a^
Other			ns
Work assessment allowance			ns
Length of relationship	0.990	[0.983, 0.997]	.004
Immigrant	0.114	[0.020, 0.649]	.014

*Note.* Multivariate logistic regression, forward stepwise (Wald). Model 4a Cox & Snell *R*^2^ = .348, Nagelkerke *R*^2^ = .465, Hosmer and Lemeshow test = .549. Model 4b Cox & Snell *R*^2^ = .375, Nagelkerke *R*^2^ = .501, Hosmer and Lemeshow test = .416. Prev. = prevalence, freq. = frequency. CI = confidence interval.

a There were no retired in the 1IPVR group and one in each of the other two groups.

b Statistical interaction between Anxiety and Childhood emotional abuse.

To attain statistical power to compare subgroups, we conducted power analyses prior to initiation of the study. The probability for the study to identify and reject the false null hypothesis (odds ratio [OR] = 1.00) was 83%.

### Ethical Aspects

The study was approved by the Regional Norwegian Ethics Committee (REK 2016/2304). All ethical and safety recommendations from the World Health Organization (WHO) were observed (WHO, 2001). An information letter informed the participants about the study objectives and that some questions were of an intimate nature. They were assured that their participation was voluntary, that they were free to withdraw from the study at any time, that withdrawal would not affect the services they received at the recruitment office, that information would be stored confidentially, and that they were welcome to call the researcher on a given phone number. All cases were included irrespective of socioeconomic status, race, ethnicity, language, nationality, sex, gender identity, sexual orientation, religion, geography, ability, and age.

## Results

In the total sample, the mean score on the Attachment avoidance subscale was 3.213 (*SD* = 1.209) and on the Attachment anxiety subscale 3.577 (*SD* = 1.080), both above the Norwegian normative mean scores. There were several significant differences between IPV victimized and nonvictimized women, and some differences between women with IPV from one and multiple partners, regarding attachment subscales and attachment subfactors in the initial bivariate analyses (Table 2). Among the 0IPVR, 37.5% had high Attachment avoidance, while 74.1% 1IPVR and 84.0% 2IPVR had high Attachment avoidance according to the Norwegian cutoff point (Olssøn et al., 2010). High Attachment anxiety was found among 66.7% 0IPVR, 74.1% 1IPVR, and 88.0% 2IPVR, and the group difference was significant on both subscales.

### Attachment Characteristics Among IPV Victimized Compared With Nonvictimized Women

The bivariate analysis showed that the IPV victimized compared with nonvictimized women had significantly or trend higher mean scores on both attachment subscales and on four out of the five attachment subfactors (Table 2). Eight sociodemographic and contextual variables showed significant group differences initially (Table 1). After multivariate logistic regression analysis of these eight (Step 2, not displayed in a table), the following two group differences remained significant: Immigrant partner and Length of relationship. In Step 3, each of the six significant or trend attachment variables was tested in a separate multivariate logistic regression model adjusted for the two significant sociodemographic variables. Three attachment variables remained significant: Attachment avoidance, Avoidance of closeness, and Uncomfortable with openness (Table 3). In Step 4, we adjusted for two childhood adversities found as risk factors in a previous part of this study: Childhood sexual abuse and Peer victimization (Ørke et al., 2020). This strengthened the model for Uncomfortable with openness (Table 3, Model 3b, Note, Cox & Snell and Nagelkerke) but not for Attachment avoidance or Avoidance of closeness. Adjusting for an interaction effect between the attachment variable and each of the two childhood adversities variables did not strengthen the models.

The strongest model showed that compared with nonvictimized women, IPV victimized women had more than three times increased likelihood of having a higher score on the Attachment avoidance subscale (Table 3, Model 1). Also, they had more than two times increased likelihood of a higher score on Avoidance of closeness (Table 3, Model 2) and on Uncomfortable with openness (Table 3, Model 3a), compared with their nonvictimized counterparts. Both Uncomfortable with openness and Childhood sexual abuse remained as independent risk factors for victimization (Table 3, Model 3b). Women victimized by IPV had more than 2.5 times increased likelihood of reporting Childhood sexual abuse (Table 3, Model 3b) compared with nonvictimized women. Two sociodemographic variables remained significant: having an Immigrant partner (Table 3, Model 1) and Length of recent relationship (Table 3, Model 1).

### Attachment Characteristics Among Women Victimized by One Compared With Women Victimized by Multiple Partners

The bivariate analysis showed that 1IPVR compared with 2IPVR had significant or trend different mean scores on both attachment subscales and on two out of the five attachment subfactors (Table 2). Among the sociodemographic and contextual variables, six showed significant or trend differences (Table 1). After a multivariate logistic regression analysis of the six sociodemographic and contextual variables (Step 2, not displayed in a table), the following two variables remained with significant group differences: how much time they generally spend on considering a new partner (Considers partner) and years of completed education (Education). On a theoretical basis, we wanted to adjust for all the significant and trend control variables. To reduce the amount of control variables to fit the sample size, we eliminated the Interpreter variable, as this is one aspect of the Immigrant variable. In Step 3, each of the four significant or trend attachment variables was tested in a separate multivariate logistic regression model adjusted for the five remaining sociodemographic variables. Only the subscale Attachment anxiety remained significant (Table 4, Model 4a).

The main finding was that women victimized by MP had a 78% increased likelihood of a higher Attachment anxiety score (Table 4, Model 4a). In Step 4, we adjusted for Childhood emotional abuse, which was found as a risk factor in a previous part of this study (Ørke et al., 2020). This did not strengthen the model. However, adding the interaction variable Attachment anxiety by Childhood emotional abuse strengthened the model (Table 4, Model 4b, Note, Cox & Snell and Nagelkerke). Childhood emotional abuse increased the association between Attachment anxiety and victimization by MP with a slightly increased likelihood (3.1%) (Table 4, Model 4b).

The control variable Work/income was not significant as such, apart from the subcategory Disability benefits (Table 4, Model 4b), indicating more recipients of disability benefits among women with IPV by MP. Furthermore, victimization by MP was associated with having a shorter relationship (Table 4, Model 4b) and being native Norwegian (Table 4, Model 4b).

## Discussion

### Main Findings

The purpose of this study was to investigate attachment differences between IPV victimized and nonvictimized women and between women victimized by one and multiple partners. We were interested in exploring whether victimization by MP increased the likelihood for certain attachment characteristics, adjusting for childhood adversities and sociodemographic variables.

The nonvictimized group scored below the normative mean regarding Attachment avoidance, whereas both victimized groups scored above the mean. Regarding Attachment anxiety, all three groups had increased Attachment anxiety scores above the Norwegian normative mean for females. These generally elevated scores may reflect a sample of only help-seeking women. Still, the three groups had significant differences among them. Our results highlight the importance of differentiating among victimized women to understand the vulnerability for IPV by MP and certain needs for this subgroup of women.

First, multivariate logistic regression analysis showed that compared with nonvictimized women, IPV victimized women had more than three times increased likelihood of a higher score on the Attachment avoidance subscale. Second, they had more than two times increased likelihood of having a higher score on both of the avoidance subfactors Avoidance of closeness and Uncomfortable with openness compared with their nonvictimized counterparts. Third, for IPV in general, Childhood sexual abuse was a significant risk factor in addition to Uncomfortable with openness. Fourth, compared with women with IPV in a single relationship, women with IPV by MP had a 78% increased likelihood of having a higher Attachment anxiety score. Finally, the association between Attachment anxiety and IPV by MP was mediated by Childhood emotional abuse, but the effect size was low.

### Attachment Characteristics Among Victimized Women Compared With Nonvictimized Women

The present study found a higher likelihood of Attachment avoidance among victimized women compared with nonvictimized women. This was nuanced by higher scores on both avoidance subcategories Avoidance of closeness and Uncomfortable with openness. The importance of Attachment avoidance was reported in some earlier studies (Kuijpers et al., 2012b; Shechory, 2013; Weiss et al., 2011; Wekerle & Wolfe, 1998) but contrasted with others reporting increased Attachment anxiety among victimized women (Bond & Bond, 2004; Lewis et al., 2017; McClure & Parmenter, 2017; Shechory, 2013). Earlier studies that did not distinguish between women with one or multiple partners may have missed important differences.

Attachment avoidance, meaning avoidance of closeness, uncomfortable with openness, distrust of partners, and deactivation of the attachment system (Feeney, 2016), may have preceded IPV victimization. IPV victimization contributed both to higher likelihood of reporting experiences of Childhood sexual abuse and higher scores on Uncomfortable with openness. These findings concur with previous findings that sexual abuse was associated with attachment avoidance (Brenner et al., 2021) and that childhood sexual abuse was associated with women’s engagement with multiple violent partners (Stein et al., 2016). A deactivating strategy associated with attachment avoidance may develop in the context of childhood sexual abuse as a way to regulate intolerable emotions, gain control over their lives, and maintain independence and a positive self-view. Velotti and coworkers (2018) suggested that avoidant individuals had typical difficulties in seeking help because of dysfunctional beliefs about being psychologically immune to emotional threats and about others being fundamentally unavailable.

However, attachment avoidance may also be a result of IPV victimization. Due to the absence of physical safety, the woman may suppress her attachment needs and withdraw to protect herself (Slootmaeckers & Migerode, 2018).

Slootmaeckers and Migerode (2018) argued that it is not simply a question of understanding individual attachment mechanisms but also the attachment dynamics of the relationship itself. Unsafe attachment and negative interaction cycles between the partners could be seen as a context that leads to IPV (Doumas et al., 2008). It was argued that in SCV the violent pursuer became aggressive to force engagement of the avoidant partner and maintain a desired level of proximity to the partner (Slootmaeckers & Migerode, 2018).

### Attachment Characteristics Among Women Victimized by One Compared With Victimized by Multiple Partners

Higher Attachment anxiety among women with IPV by MP involves excessive need for approval, fear of abandonment, and distress and hurt in the face of conflict (Feeney, 2016). Our result deviated from Alexander’s finding (Alexander, 2009) of unresolved attachment style. The reason for the divergent findings may be that these two studies applied measurements from two different approaches wherein the attachment concepts are not operationalized in the same way.

While some studies have reported increased attachment anxiety among IPV victimized in general (Bond & Bond, 2004; Lewis et al., 2017; McClure & Parmenter, 2017; Shechory, 2013), the contribution from the present study was that increased Attachment anxiety characterized women victimized by MP in particular.

A statistically significant interaction variable of Attachment anxiety by Childhood emotional abuse increased the likelihood for IPV by MP, suggesting that experiences of Childhood emotional abuse increased the association between higher Attachment anxiety score and victimization by MP. This was supported by Valdez and colleagues (2013), who reported a childhood emotional trauma trajectory associated with a desire for intimacy, fear of loneliness, IPV, and deficits in navigating interpersonal relationships. Having grown up with humiliating and invalidating caregiving, this group of women with increased attachment anxiety may view adult relationships as opportunities for acceptance. A propensity to seek closeness and ingratiate themselves with their partners (Downey & Feldman, 1996) may prevent their recognition of a partner’s early risk behaviors, putting themselves at risk for further maltreatment (Hocking et al., 2016).

Compared with nonvictimized women, victimized women had higher Attachment avoidance scores. Compared with 1IPVR, women with IPV by MP had higher Attachment anxiety scores. To speculate, our findings indicated that compared with nonvictimized women, women with IPV by MP may possibly display a mixed attachment strategy with higher scores on both Avoidance and Anxiety dimensions than nonvictimized women do. This might have an especially destructive effect, possibly trapping the women in a cycle of conflict-riddled attempts to meet personal needs while trying to avoid rejection and mishandling (Mikulincer & Shaver, 2016).

It has been hypothesized that high levels of attachment anxiety among victims of IPV may make it more difficult to leave an abusive relationship (Mikulincer & Shaver, 2016; Park, 2016). As described initially, attachment anxiety involves excessive need for approval from others, fear of interpersonal rejection or abandonment, and distress when one’s partner is unavailable or unresponsive (Cassidy & Shaver, 2016; Pedersen et al., 2015). In the present study, there was no measure of the act of leaving, but of relationship length. The results showed that the group of women with IPV by MP in particular exhibited the highest levels of Attachment anxiety and reported shorter relationships. Higher levels of Attachment anxiety seemed, in this case, to contribute to a higher likelihood of engaging in short, destructive relationships rather than long relationships. Including the result that victimized women in general had more than three times increased likelihood of higher score on Attachment avoidance (Table 3) may inform this finding. Attachment avoidance involves need for self-reliance and fear of interpersonal closeness (Cassidy & Shaver, 2016; Pedersen et al., 2015). Shorter, destructive relationships among women with IPV by MP may follow a combination of high levels of both attachment anxiety and attachment avoidance in this group.

The increased attachment scores could have preceded IPV due to childhood trauma or they could have been reinforced by the current IPV (Alexander, 2009). Slootmaeckers and Migerode (2018) suggested a pattern of SCV, which had its origins in a negative interaction cycle of clinging and withdrawal. Violence was seen as an attempt to regulate distance from the continuous contact-seeking of the clinging partner (Slootmaeckers & Migerode, 2018). While yearning for contact, the clinging partner was pushed aside and may in turn seek even more comfort, connection, and proximity. Due to their heightened sense of insecurity, the clinging partners became increasingly overwhelmed by powerlessness and separation anxiety (Slootmaeckers & Migerode, 2018). Doumas and colleagues (2008), too, reported that the “mispairing” of an avoidant male partner with an anxious female partner was associated with both male and female violence. However, when controlling for partner violence, the relationship between attachment and violence was significant for males only (Doumas et al., 2008).

A recent longitudinal study reported that attachment anxiety was associated with increased risk for experiencing physical assault, while attachment avoidance was unrelated to subsequent IPV victimization (Sandberg et al., 2019). To measure causality one must have a prospective design. Therefore, our results do not provide causality between attachment avoidance and IPV or between attachment anxiety and IPV by MP in particular.

Most research on adult attachment was based on the assumption that working models are relatively general and trait-like. Recent research, however, suggests that people develop attachment representations that are relationship-specific, leading people to hold distinct working models in different relationships (Fraley et al., 2011). Slootmaeckers and Migerode (2018) argue that the attachment pattern in a given romantic relationship is the result of attachment disposition (childhood), past romantic attachment, and contemporary interaction and experience with this partner. Dispositional attachment and situational attachment interact (Slootmaeckers & Migerode, 2018).

A person’s position on the Anxiety and Avoidance dimensions can move across different, temporarily separated assessments, partly due to contextual factors, partly due to normal measurement error, and partly due to real change over time (Mikulincer & Shaver, 2016).

In addition to applying the frequently used subscales Attachment avoidance and Attachment anxiety, this study explored the application of five subfactors (Pedersen et al., 2015). Comparing victimized with nonvictimized women, the strongest model showed that the subfactor Uncomfortable with openness and Childhood sexual abuse were separate risk factors for IPV. Adding Childhood sexual abuse did not contribute to substantial changes of the OR value for Uncomfortable with openness (Table 3, Model 3b). Except for this, we found that the application of the five subfactors did not add substantially to the results.

### Limitations

Some young participants may be early in their victimization “career” and would later in life appear in the IPV by MP group, and this may blur group differences.

Discussing results regarding attachment is complicated due to research traditions applying different methodologies. The concepts of attachment anxiety and avoidance are not operationalized in the same way in the developmental and the social approach. There are more measures than constructs, and the measures do not necessarily correspond with each other or with any particular understanding of the construct (Mikulincer & Shaver, 2016). Furthermore, studies regarding categories of attachment styles may give a different picture than studies of scores along attachment scales.

As found in several studies of help-seeking women after IPV, a considerable number of the invited women declined to participate. We have no information regarding these women concerning group differences. Therefore, an analysis of the representativeness of the study sample was not possible. We may have missed women who declined participation due to health problems, social distress, or other difficulties. The experiences of these women might have differed from those of the included women. Another important limitation is that the present study only included information about help-seeking women. They may differ in several ways from women who are not seeking help (Dufort et al., 2013). Thus, findings from this study of help-seeking women do not necessarily generalize to all help-seeking victims, to victimized women who do not seek help, to community samples, or to women outside of Norway, due to cultural, social, and societal differences. Cultural context is important in understanding IPV risk markers (Mallory et al., 2016). This calls for careful interpretation of the generalizability of our findings.

Some of the ORs were high. Still, wide confidence intervals regarding Immigrant partner and Disability benefits indicate that these findings should be interpreted with caution.

Finally, the cross-sectional design has limitations concerning any assumptions of causality and temporal ordering of variables.

### Clinical Implications

The results suggested that women victimized by MP had specific attachment issues: high on avoidance and high on anxiety. Accordingly, all IPV victimized women would not benefit from the same treatment. Victimized women should be assessed regarding attachment anxiety and avoidance, and childhood sexual and emotional abuse.

Women at increased risk might benefit more from long-term intervention. They should be invited step-by-step to talk about these topics in therapy and might be guided toward an increased awareness of how attachment issues have affected their relationship (Velotti et al., 2018). Therapy should target fears of rejection and excessive need of approval in relation to the choice of a new partner. Clinicians might help with developing skills, so that when attachment anxiety or avoidance is triggered, clients are less likely to react automatically and more likely to respond consciously and constructively in ways that do not compromise their dignity and well-being (Park, 2016). Improved insight in these therapy topics may inform the women to engage in the prevention of future IPV relationships.

Focusing on the discrepancy between partners’ needs for intimacy and distance within the couple has been suggested as a strategy for treating IPV (Doumas et al., 2008). Emotionally focused therapy (EFT) emphasizes emotions and attachment (S.M. Johnson, 2007). Based on EFT, it was argued that negative interaction cycles may be discussed with couples suffering from SCV, but not intimate terrorism (IT), when ethics and safety allow (Slootmaeckers & Migerode, 2018). However, it is important to keep in mind that very few risk factors establish a causal relationship (Park, 2016).

### Research Implications

More research is needed to investigate the interaction between increased attachment anxiety and IPV by MP; the temporal order of the variables is yet to be described, as well as whether increased attachment anxiety is possibly disturbing the initial process of partner choice or the dynamics within the relationship. Speculations regarding a combination of increased attachment anxiety and avoidance among women with IPV by MP would require further empirical investigation. Moreover, differentiating between Johnson’s types of violence (M. P. Johnson, 2008) may help nuance the association between attachment style and risk of IPV revictimization by MP.

## Conclusion

In this study, we found differences in attachment characteristics both between women victimized by one and multiple partners, and between victimized and nonvictimized women. The results supported the relevance of attachment theory for understanding IPV victims. Both attachment anxiety and attachment avoidance appeared influential in IPV by MP. The findings suggested that interventions should especially reach multiply victimized women with high attachment anxiety before initiation of future intimate relationships.
